# Supplementary material for: Efficacy and Safety of Visible and Near-Infrared Photobiomodulation Therapy on Astenospermic Human Sperm: Wavelength-Dependent Regulation of Nitric Oxide Levels and Mitochondrial Energetics
Source: Biology (Basel). 2025 May 1;14(5):491. doi: 10.3390/biology14050491 (PMC12109510; doi:10.3390/biology14050491)
Supplement: Supplementary file 1 [file biology-14-00491-s001.zip › biology-3586324-supplementary.pdf]

Table S1. Semen temperature variations at the end of irradiation. The OMEGA thermal monitoring system performed measures.

	450 nm	635 nm	810 nm	940 nm	1064 nm
0 W (0 J)	37.0	37.0	37.0	37	37.0
0.25 W (15 J)	37.5	37.0	37.0	38.5	38.0
0.50 W (30 J)	38.5	37.0	37.0	39.5	39.0
1.00 W (60 J)	40.0	37.5	37.5	41.5	41.0
2.00 W (120 J)	42.0	39.5	39.0	44.0	43.0

White cells represent controls. Green cells indicate wavelength/energy parameter combinations that increase sperm energetic activity (ATP/AMP ratio, Fig. 6.). Red cells denote inhibition of energetic activity. In contrast, yellow cells indicate no effect compared to the control.
